# Supplementary material for: Complexome profiling of the Chlamydomonas psb28 mutant reveals TEF5 as an early PSII assembly factor
Source: Plant Cell. 2025 Mar 17;37(6):koaf055. doi: 10.1093/plcell/koaf055 (PMC12164590; doi:10.1093/plcell/koaf055)
Supplement: koaf055_Supplementary_Data [file koaf055_supplementary_data.zip › psb28_interactive.zip!/plots/intensity/qualityPlots/peptides/CLV2_Cre17.g729450.t1.2.html]

### Cre17.g729450.t1.2

Trivial name: CLV2  
SeqCoverage [median]: 2.10  
Mass: 99.49 kDa  
  
 

| Sequence | MissedCleavage | Mass [Da] | wtIntensity | muIntensity | Score |
| --- | --- | --- | --- | --- | --- |
| ILEMMR | 0 | 791.40 | 0.00 | 407.15 | 58.66 |
| VSESLDYEPIQNK | 0 | 1520.74 | 0.00 | 485.74 | 77.38 |
|  |  |  | 0.0 | 892.9 |  |
